# Development and Preliminary Validation of a New Type 1 Diabetes Adjustment Scale (DAS-1)

**DOI:** 10.3389/fpsyg.2020.00533

**Published:** 2020-04-16

**Authors:** Teresa Rivas, Mónica Carreira, Marta Domínguez-López, Maria Soledad Ruiz de Adana, María Teresa Anarte

**Affiliations:** ^1^Department of Psychobiology and Behavioral Sciences Methodology, University of Málaga, Málaga, Spain; ^2^Department of Personality, Assessment and Psychological Treatment, Institute of Biomedical Research of Malaga (IBIMA), University of Málaga, Málaga, Spain; ^3^Diabetes Unit, Department of Endocrinology and Nutrition, Institute of Biomedical Research of Málaga (IBIMA), Regional University Hospital of Málaga, University of Málaga, Málaga, Spain; ^4^CIBERDEM, Madrid, Spain

**Keywords:** type 1 diabetes, adjustment scale, assessment, robust factor structure, reliability, validity

## Abstract

**Background:**

This study focuses on the development and validation of a new Type 1 Diabetes Adjustment Scale (DAS-1).

**Method:**

A total of 204 participants aged 15–65 with type 1 diabetes completed the self-report measures of the DAS-1, which includes clinical and psychological variables.

**Results:**

Robust confirmatory factor analysis detected a unidimensional structure of the item scores. The omega coefficient was 0.91 and test–retest reliability was 0.87. Classifying subjects as in a Positive or Negative mood state, ROC analysis yielded an optimal cut-off of 50 for the DAS-1 scores, with a clinical accuracy of AUC = 0.85. The DAS-1 demonstrated evidence of good reliability and acceptable construct validity.

**Conclusion:**

The DAS-1 demonstrated good clinical utility, good sensitivity and adequate specificity. Clinical and theoretical implications of these results are discussed.

## Introduction

Scientific interest in understanding how psychological factors may determine medical conditions is growing. Psychological aspects may seriously impact somatic symptoms and medical outcomes, especially in chronic diseases (Martino et al., 2019). After the initial impact of the diagnosis of a chronic disease such as type 1 diabetes (T1D), an adjustment period begins in which the individual must learn to live with the disease, which implies that these patients must take actions to become accustomed to their new circumstances (de Ridder et al., 2008). A diagnosis of T1D also leads to deterioration in the patient’s health. Some authors (Isla Pera et al., 2008) who have studied adaptation to T1D have considered the process to be equivalent to the mourning process described by Kübler-Ross (1993), which includes the following stages: denial, rebellion, negotiation, depression and acceptance. In the case of T1D this latter stage would be the adaptation to the disease involving a relative sense of well-being and balance. If the process concludes positively, patients with T1D are able to adapt to the new situation while maintaining quality of life. However, if the adaptation process does not occur or is inappropriate, these patients will find that their quality of life as well as their psychological and physical well-being will be affected (Malik and Koot, 2009). This perception of balance is unstable and can be disrupted by various situations such as personal or family issues or problems produced by T1D. Consequently, the person with T1D will have to achieve a new balance to adjust to the disease throughout his or her life. “Adjustment to diabetes” is therefore understood as the healthy rebalancing that the patient must undergo to the new circumstances of diabetes (de Ridder et al., 2008).

Factors including the demands of treatment, the impact of the diagnosis, uncertainty about possible complications and frustration with unexplained blood glucose levels or lifestyle restrictions (Anarte, 2004) can trigger the onset of negative emotions. These negative emotions can lead to elevated depressive symptoms (Robertson et al., 2012), making it difficult for the patient to adapt to diabetes. In addition, because T1D is a chronic disease, adjustment to it can change over a patient’s life course at different times such as when learning to self-manage the disease, during life transitions, impacting disease self-management, disease progression, and the onset of complications (Young-Hyman et al., 2016).

Other factors associated with diabetes treatment adherence may influence patient adjustment to T1D such as coping styles, depressive symptoms, perceived social support, and diabetes-related distress (Di Matteo, 2004; Ortiz, 2006; Sacco and Yanover, 2006; Fisher et al., 2014). These studies suggest it is difficult to achieve adequate glycemic control due to the influence of these variables.

Personal resources such as coping strategies produce improved adjustment to T1D. Greater psychological adjustment has been found in those patients who use strategies focused on the problem versus other strategies (Duangdao and Roesch, 2008), showing an increase in well-being, mood and self-esteem. Other authors agree that problem-focused coping is more beneficial to the patient’s health than emotion-focused coping (Frazier, 2000). The study of coping in T1D was of great interest after Peyrot et al. (1999) reported that emotion-centered coping and problem-centered coping may be associated with glycemic control. Similarly, Ortiz (2006) associates better metabolic adjustment with behavioral coping than with emotional coping, while Boland and Grey (1996) find that emotion-centered coping is associated with poor metabolic control, including nonadherence to the therapeutic regimen.

Psychosocial factors play an integral role in managing diabetes but differ according to the type. T1D and Type 2 diabetes (T2D) are distinct conditions that have different psychological effects. Delamater et al. (2001) have reported how Psychosocial functioning, Neurocognitive functioning, Psychosocial factors associated with regimen adherence, metabolic control, and quality of life are affected. Neurocognitive deficits have been observed in adults with T1D, particularly those with at least five episodes of severe hypoglycemia, and in patients with peripheral neuropathy. Among older adults with T2D, cognitive deficits have been reported in association with poor glycemic control. More recently, it has been found that adults with T1D report feeling judged negatively for not managing their diabetes “perfectly” (Browne et al., 2014) while adults with T2D report feeling blamed and shamed for “bringing the condition on themselves” (Browne et al., 2013).

It would appear that both types of diabetes need to be assessed with different instruments (Polonsky et al., 2005). Indeed, a number of specific instruments have been developed to assess quality of life (Bott et al., 1998), distress (Polonsky et al., 2005; Fisher et al., 2015), or confidence in diabetes self-care (Van Der Ven et al., 2003; Polonsky et al., 2009). Although several instruments have been developed to assess adjustment to diabetes, they have some limitations. Some evaluate acceptance either to disease in general (Linkowski, 1971) or to diabetes (Schmitt et al., 2018), and others assess adjustment in adolescents (Sullivan, 1979; Wysocki et al., 1992), emotional adjustment to diabetes (Deleon, 1995) or adjustment in patients with T2D (Ebrahimi et al., 2016). However, none of these instruments specifically evaluate adjustment to diabetes in adults with T1D. In addition, the concept of adjustment is different in each of these. For these reasons, the aim of this study was to develop a new instrument specifically designed to provide a reliable and valid measurement of adjustment to T1D in adults.

## Materials and Methods

### Participants

A total of 204 participants with T1D were included (48% men and 52% women), with a mean age of 33.82 years (*SD* = 11.09; Range: 15–65), from the Diabetes Unit (Department of Endocrinology and Nutrition, Regional University Hospital of Malaga, Spain) between 2009 and 2012. Women with gestational diabetes were excluded. The mean number of years with T1D was 15.16 (*SD* = 9.64; Range: 0.04–50), and mean glycosylated hemoglobin (HbA_1c_) was 7.61% (*SD* = 1.41; Range: 5.10–14.00%). Additional sample characteristics are shown in Table 1.

**TABLE 1:** Characteristics of the study sample.

Variable	*n* (%)	*M (SD)*	DAS-1 scale scores *M (SD)*
**Diabetes complications**			
Yes	37 (18.1)		45.86 (15.86)
No	167 (81.9)		40.40 (11.50)
**Other chronic diseases**			
Yes	60 (29.6)		45.11 (14.22)
No	143 (70.4)		39.79 (11.50)
**Family support**			
Yes	193 (95.1)		40.78 (12.26)
No	10 (4.9)		54.30 (11.48)
**Partner support**			
Yes	156 (76.8)		40.48 (11.89)
No	47 (23.2)		44.66 (14.17)
**Work support**			
Yes	141 (69.5)		39.55 (10.99)
No	62 (30.5)		45.77 (14.71)
**Personal health support**			
Yes	191 (94.6)		40.88 (11.82)
No	11 (5.4)		52.09 (19.65)
**Body Mass Index (BMI, Kg/m^2^) +**			
<30 normal weight/overweight	174 (88.32)		40.64 (11.68)
≥30 ∩ <34.2 obese	19 (9.64)		41.00 (11.10)
≥34.2 ∩<43.9 extreme obese(^∗^)	4 (2.04)		42.50 (13.96)
**HbA_1c_ (%)**			
≤7 good metabolic control	80 (39.6)		40.51 (11.09)
>7 ∩<10 poor metabolic control	113 (55.9)		40.93 (13.25)
≥10 extremely poor metabolic control (^∗^)	9 (4.5)		52.56 (6.39)
Hypoglycemic episodes (week) (*n* = 202)		2.41 (2.40)	
Hyperglycemic episodes (week) (*n* = 203)		3.32 (3.39)	
Self-monitoring (*n* = 203)		3.89 (1.77)	
Age		33.82 (11.09)	
Number of years with diabetes		15.16 (9.64)	
MST		2.13 (0.86)	
TCD		2.37 (1.06)	
SKD		2.12 (0.80)	
SCD		3.42 (1.10)	
DQOL-Dissatisfaction score		33.34 (9.49)	
DQOL-Impact score		32.54 (8.79)	
DQOL-Social Concern score		14.04 (5.49)	
DQOL-Concern for the future score		9.37 (3.34)	
DQOL score		89.31 (23.02)	
BDI-II score		9.73 (10.09)	
SDS score		38.08 (11.00)	
STAI-S score		20.07 (12.95)	
STAI-T score		20.08 (12.06)	

MST, mood state; TCD, satisfaction with time to control diabetes; SKD, satisfaction with the knowledge of diabetes. SCD, worried about experiencing complications due to diabetes; DQOL, diabetes quality of life scale; BDI-II, beck depression inventory. SDS, self-rating depression scale; STAI- S, state-trait anxiety inventory-state; STAI- T, state-trait anxiety inventory-trait. (*) Extremely obese and extremely poor metabolic control groups determined using the (Q3+1.5 × IQR) rule for possible outliers.

### Measures

#### Development of the Self-Rating Diabetes Adjustment Scale for Type 1 Diabetes (DAS-1)

The items were developed based on the factors that influence patient adjustment to diabetes according to the scientific literature. Patients encounter *barriers* in diabetes self-management or in their lifestyle (Interference: items 6,8,9,10,11,12,13). People with diabetes often feel frustrated, tired or overwhelmed by the demands of diabetes (Rubin, 2005), which affect its course. The demands of T1D treatment and the delicate balance that patients must maintain draw on all their resources, often triggering the onset of *negative emotions* (Negative emotions: items 1, 4, 5), such as distress or depression (Anarte, 2004; Peyrot et al., 2005; Lloyd et al., 2010). *The impact of diabetes on patients and their relatives (family)* can be important (Diabetes Impact: items 2,3,7,15). Indeed, this stage is related to nonclinical adjustment behaviors and symptoms in the subjects (Young-Hyman et al., 2016). The patient’s adjustment to the new situation depends considerably on the *coping strategies* used to manage the disease (Coping style: items 14,16,19,20). As indicated above, the type of coping used will determine better or worse psychosocial adjustment and glycemic control (Duangdao and Roesch, 2008; Jaser et al., 2012). *Treatment adherence* is a basic pillar for good control of T1D. Some patient behaviors, such as avoidance, may interfere with treatment adherence behaviors (items 17,18), which would imply worse glycemic control and poorer self-care (Jaser et al., 2012).

Based on theoretical background, a pool of 20 items (Table 2) was selected by consensus among four external experts (two psychologists, specialized in health psychology and diabetes, and two endocrinologists). Individuals with T1D (*N* = 25) completed the 20 items in the presence of a psychologist and were allowed to ask questions. It was determined whether the item was properly understood or was more understandable using different wording. Finally, the item pool was reviewed by all the authors to identify redundancy, language comprehension and other issues. The DAS-1 is a visual analog, numerical rating scale. Items are rated on a five point scale from 1 (not at all) to 5 (extremely). A higher score on the DAS-1 scale indicates poorer patient adjustment to the disease.

**TABLE 2:** Type 1 diabetes adjustment scale (DAS-1): items scale.

(1) Are you experiencing a stressful situation that is affecting you?
(2) Are you angry about your diabetes?
(3) Are you more nervous since you have been diagnosed with diabetes?
(4) How often do you have a feeling of emptiness or sadness?
(5) How often do you have negative thoughts about yourself and your future?
(6) Do you feel limited by your diabetes?
(7) How often do you think diabetes is burdensome?
(8) How much does your diabetes interfere in your family life?
(9) How much does your diabetes interfere in your sexual life?
(10) How much does your diabetes interfere in your job?
(11) How much does your diabetes interfere in your social life?
(12) How much does your diabetes interfere in your physical appearance?
(13) How much does your diabetes interfere in your future?
(14) To what extent has dealing with diabetes helped you to grow as a person?
(15) I know I have diabetes and I accept it.
(16) Although I know what to do in order to treat my diabetes, I don’t want to do it.
(17) I focus on my job and other activities in order to not think about my diabetes.
(18) When I consume alcohol and other substances, I’m better.
(19) Talking to my family and friends about my diabetes usually makes me feel
better.
(20) I prefer not to think I have diabetes.

### Sociodemographic Variables

In order to study the sociodemographic variables of the patients, a structured interview was administered during the evaluation period. Age and number of years with diabetes were collected and used as continuous variables.

### Biomedical Variables

For the collection of the biomedical data, medical staff completed a structured interview with each patient, recording the following variables:

- Dichotomous response variables (Yes/No): diabetes complications; other chronic diseases; and family, partner, work, and personal health support.
- Categorized response variables: body mass index (BMI), HbA_1c_ (%).
- Discrete response variables: number of hypoglycemic/hyperglycemic episodes per week, frequency of daily self-monitoring.

Body mass index was calculated using the formula: weight/height^2^. Glycosylated hemoglobin (HbA_1c_), measured by high pressure liquid chromatography with a Kyoto Daiichi Kagaku device, was used as an indicator of metabolic control.

### Mood, Satisfaction and Concern About Diabetes

Variables were measured on a 5-point scale from 1 (not at all) to 5 (extremely) using the following questions: *Mood State (MST)*: In general, what is your mood state? *Satisfaction with time to control diabetes (TCD)*: Are you satisfied with the amount of time you take in controlling your diabetes? (1 Very much to 5 Very little). *Satisfaction with the knowledge of diabetes (SKD)*: Are you satisfied with your knowledge about diabetes? (1 Very much to 5 Not at all). *Worried about experiencing complications due to diabetes (SCD)*: Are you worried about experiencing complications because of diabetes in the future? (1 Very much to 5 Not at all).

### Diabetes Quality of Life (DQOL)

The DQOL (The Diabetes Control and Complications Trial Research Group [DCCT], 1988) measures the quality of life of persons with diabetes. The Spanish version (Millán et al., 2002) consists of 43 items that form four dimensions: Satisfaction with treatment, Impact of treatment, Concern regarding social and vocational aspects and Concern regarding the future effects of diabetes. Items are rated on a 5-point Likert-type scale (1–5) with lower scores indicating higher quality of life. Cronbach’s α values for the four subscales were 0.87, 0.85, 0.80, and 0.75, respectively.

### Beck Depression Inventory (BDI-II)

This instrument evaluates the intensity of the depressive symptoms that an individual presents. This self-administered questionnaire is composed of 21 multiple-response items (0, 1, 2, 3), according to the severity of the symptom (from 0, which indicates the absence of the symptom, to 3, which represents the maximum severity of the symptom). The Spanish adaptation of the BDI-II (Beck et al., 1996) by Sanz et al. (2005) was used. Cronbach’s α value was 0.94.

### Self-Rating Depression Scale (SDS)

The SDS (Zung, 1965) is composed of 20 items of which two are affective, eight refer to somatic correlates and 10 to psychological correlates (10 positive and 10 negative). The items are assessed on a scale of 1–4 points concerning the frequency with which each behavior included in the scale occurs. The internal consistency in the Spanish adaptation (Conde, 1969) was 0.88.

### The State-Trait Anxiety Inventory (STAI)

*To evaluate anxiety*, the Spanish adaptation of the STAI (Spielberger et al., 1982) by Seisdedos (1988) was used. It comprises two self-assessment scales with 20 items rated on a Likert-type scale from 0 to 3: the *State Anxiety Inventory* (STAI-S) and the *Trait Anxiety Inventory* (STAI-T). The STAI-S assesses the subject’s status when faced with threatening situations at a given time, while the STAI-T assumes a permanence of anxiety in the subject, evaluating the disposition to respond with high rates of anxiety to stressful situations. Cronbach’s α values were 0.92 and 0.95, respectively.

### Procedure

This study was evaluated by the Research and Ethics Committee of the Regional University Hospital of Malaga and received a positive assessment. Consent was obtained from all participants after they were informed about the purpose of the study and the voluntary nature of their participation. The sociodemographic and clinical variables of the participants were collected through a structured interview together with the psychological variables by a clinical psychologist in an examination room of the Diabetes Unit during a visit with the endocrinologist. The participants completed the 20-item DAS-1, the questionnaires and the variables described above. To explore the test–retest reliability of the DAS-1, 36 randomly selected patients completed all the DAS-1 items twice, over a mean interval of 2 weeks. The participants did not receive economic compensation.

### Data Analysis

Preliminary analyses with univariate descriptive statistics of the items (mean, standard deviation, skewness and kurtosis, outliers) were conducted. The definition used for an outlier was based on Moore et al. (2009). An observation was a suspected outlier when it fell more than 1.5 × the Interquartile Range (IQR) above the third quartile or below the first quartile. Kolmogorov–Smirnov tests for univariate normality and Mardia’s test for multivariate normality (Mardia, 1970) were also performed.

Parallel Analysis (Horn, 1965) and Very Simple Structure (VSS) (Revelle and Rocklin, 1979) were carried out to explore the number of underlying dimensions. As these indices suggested, a one-factor model was tested. Robust Confirmatory Factor Analysis (RCFA) and a Robust Weighted Least Squares estimation method for categorically ordered data were conducted using a polychoric correlation matrix. Goodness of fit was evaluated using the following indices: relative chi-square ratio (χ^2^/*df*), standardized root mean square residual (SRMR), root mean square error of approximation (RMSEA), the comparative fit index (CFI), the Tucker-Lewis index (TLI), and the weighted root mean residual (WRMR). Model fit was assessed by the following criteria: For a good fit model, the ratio χ^2^/*df* should be as small as possible. A ratio 0≤χ^2^/*df*≤2 is indicative of a good fit and 2 < χ^2^/*df*≤3 for an acceptable data-model fit (Schermelleh-Engel et al., 2003). The 0≤*RMSEA*≤0.05 value is considered a good fit, 0.05 < *RMSEA* < 0.10 an acceptable fit, and *RMSEA*≥0.10 a poor fit (Browne and Cudeck, 1993). The 0≤*SRMR*≤0.05 is considered a good fit, whereas values 0.05 < *SRMR*≤0.10 may be interpreted as an acceptable fit (Hu and Bentler, 1995). The CFI and TLI values should be greater than or close to 0.90 for a good fit (Hopwood and Donnellan, 2010). Yu and Muthén (2002) recommend the WRMR over the SRMR for categorical indicators, with good fit at values close to and below 1.00.

Internal consistency was analyzed with omega (ω) (McDonald, 1999) and item analysis was calculated with ω deleting each item in turn. The 95% confidence intervals (CI) for ω and ω (-item) were also calculated. Test–retest reliability was obtained using the Interclass Correlation Coefficient (ICC). ICC values less than 0.50, between 0.50 and 0.75, between 0.75 and 0.90, and greater than 0.90 are indicative of poor, moderate, good, and excellent reliability, respectively (Koo and Li, 2016).

The criterion validity of the DAS-1 scores for the variables MST, TCD, SKD, and SCD was analyzed using Pearson’s correlation coefficients. According to Evers et al. (2013), a criterion validity value can be considered Inadequate (*r* < 0.20), Adequate (0.20 _≤_ *r* < 0.35), Good (0.35 _≤_ *r* < 0.50) or Excellent (*r*_≥_ 0.50). Receiver operating characteristics (ROC) curve analysis was conducted to determine the optimal cut-off value for the DAS-1 scores (Zweig and Campbell, 1993; Bonillo et al., 2000) for differentiating between the established Positive (PMS) or Negative (NMS) mood state of the criterion groups, dichotomizing by the MST mean. Other criterion validity indices were evaluated using Pearson’s correlation between the DAS-1 and measures of quality of life, depression and anxiety.

Construct validity evidence was examined with Pearson’s correlation between the DAS-1 scale scores and the clinical variables (number of hypoglycemic/hyperglycemic episodes per week, frequency of daily self-monitoring), age, and number of years with diabetes. Interpretation of Pearson’s correlation was according to the criteria of Evers et al. (2013). Additional *construct validity evidence* was analyzed using Student-Welch’s *t*-test comparing DAS-1 means in groups defined by clinical variables including diabetes complications, other chronic diseases, and family, partner, work or personal health support. The effect size of the mean difference (*d*), power (1-β) and 95% CI were also calculated. DAS-1 median comparison was performed using the Kruskal–Wallis test in groups defined by HbA_1c_ and BMI. These analyses were performed using different R packages [*psych* version 1.7.3.21 (Revelle, 2015), *paran* version 1.5.1 (Dinno, 2009), *MBESS* version 4.2.0 (Kelley, 2007), *lavaan* 0.5–12 (Rosseel, 2012)] and IBM SPSS Statistics, version 14.0.

## Results

### Preliminary Analysis

Item descriptive statistics are shown in Table 3. All items ranged from 1 to 5. Item skewness values ranged from 0.20 to 2.36. There were six items with skew greater than one. Item kurtosis values were between −1.37 and 6.03, with item 18 showing a highly leptokurtic distribution. The number of suspected outliers of items based on the 1.5 x IQR rule are shown in Table 3. The significant Kolmogorov–Smirnov test (*p* < 0.001) indicated that the answers to each item did not satisfy normality.

**TABLE 3:** Item descriptive statistics, ω and 95% CI ω indices.

							ω if item is
					Number of	ω if item	deleted
Item	*M*	*SD*	Skew	Kurtosis	Outliers	is deleted	95% CI
1	2.57	1.41	0.20	–1.37	0	0.91	(0.88, 0.93)
2	2.06	1.04	0.89	0.26	0	0.90	(0.87, 0.92)
3	2.03	1.04	0.79	–0.09	0	0.90	(0.87, 0.92)
4	2.36	1.04	0.46	–0.57	4	0.90	(0.87, 0.92)
5	2.36	1.08	0.36	–0.71	0	0.90	(0.87, 0.92)
6	2.35	1.03	0.36	–0.39	6	0.90	(0.87, 0.92)
7	2.61	1.21	0.32	–0.80	10	0.89	(0.87, 0.92)
8	1.80	0.94	0.98	0.29	10	0.90	(0.88, 0.92)
9	1.67	0.87	1.23	0.97	9	0.90	(0.88, 0.92)
10	2.09	1.02	0.58	–0.56	0	0.90	(0.88, 0.92)
11	1.63	0.86	1.31	1.15	8	0.90	(0.88, 0.92)
12	1.79	0.95	0.95	0.08	2	0.90	(0.88, 0.92)
13	2.29	1.07	0.57	–0.42	0	0.90	(0.87, 0.92)
14	2.42	1.11	0.67	–0.13	10	0.91	(0.88, 0.93)
15	1.63	0.86	1.62	2.80	9	0.90	(0.88, 0.92)
16	2.07	1.11	0.73	–0.40	0	0.90	(0.88, 0.93)
17	1.76	1.01	1.35	1.26	10	0.90	(0.88, 0.93)
18	1.31	0.67	2.36	6.03	10	0.90	(0.88, 0.93)
19	2.68	1.39	0.33	–1.14	0	0.91	(0.89, 0.93)
20	1.91	1.18	1.28	0.73	10	0.90	(0.88, 0.92)
Scale	41.39	12.54	0.71	0.61	2	0.91	(0.88, 0.93)

There was no multivariate normality [Mardia’s skewness and kurtosis tests were significant, *p* < 0.001 with χ^2^(*Skew*) = 3008.23(88.48) and *Z*−*value*(*Kurtosis*) = 19.05(519.15)]. All correlations were below 0.78, indicating no multicollinearity in the data.

In the study of underlying dimensionality, Horn’s parallel analysis (mean = 3.29) and VSS Complexity 1 (maximum = 0.97) suggested a one-factor solution.

### Robust Confirmatory Factor Analysis

**FIGURE 1:**
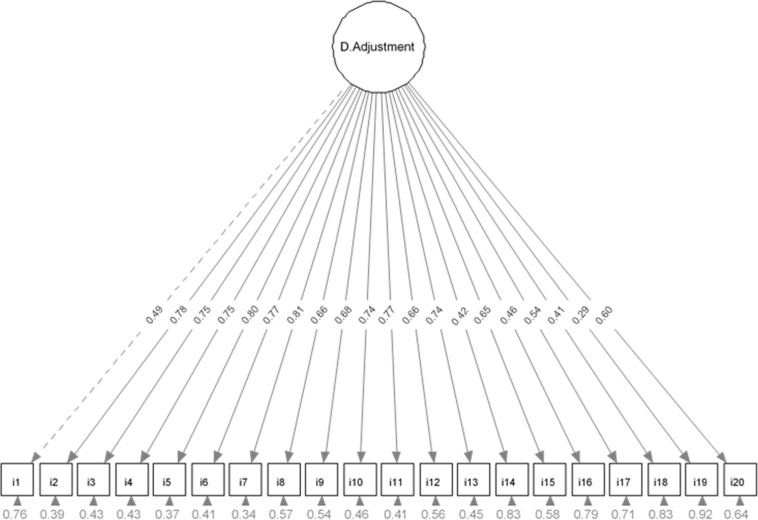
Path diagram of the Robust Confirmatory Factor Analysis.

A one-factor model was tested using RCFA. Figure 1 shows the corresponding path diagram with standardized loadings and estimated variances of the 20 DAS-1 items. All loadings exceeded the threshold 0.40 with the exception of item 19 (0.29) and all were significant (*p* < 0.001). The estimated item variances were >0.40 except for i2 (0.39), i5 (0.37), and i7 (0.34). Robust goodness of fit indices were: χ^2^/*df* = 2.69, *RMSEA* = 0.09 (90% CI: 0.08–0.10), *SRMR* = 0.08, *WRMR* = 1.13, *CFI* = 0.93, and *TLI* = 0.92. These results indicate an acceptable fit, and this dimension was named Adjustment to Diabetes.

### Reliability and Item Analysis

*Internal Consistency* of the DAS-1 was ω (0.91) and ω 95% CI [0.88, 0.93]. ω if each item is deleted ranged from 0.89 to 0.91. ω if each item is deleted was lower than 0.91, except for ω if item 19 is deleted being equal to 0.91, indicating that no item should be removed from the scale. These indices and their 95% CI are shown in Table 3. All 20 items are considered in the following analyses.

#### Test–Retest Reliability

The DAS-1 scores in Time-1 (*M* = 40.11, *SD* = 13.16, *N* = 36) and Time-2 (*M* = 39.33, *SD* = 14.75, *N* = 36) showed an *ICC* = 0.87.

The DAS-1 is a unidimensional scale with an acceptable fit by RCFA, has an excellent internal consistency (≥ 0.90) (Evers et al., 2013), and good test–retest reliability. The DAS-1 total score distribution showed a moderate skew (0.50 < skew < 1), to be slightly leptokurtic and with two outliers (Table 3).

### Criterion-Related Validity

The variables MST, TCD, SKD, SCD have low or moderate skewness values (<1) and low kurtosis values with MST and SKD having fewer outliers, one and zero, respectively (Table 4). These variables did not indicate a strong deviation from the normal distribution in the sample, but the Kolmogorov–Smirnov normality test was significant (*p* < 0.001) for all the variables. Pearson’s correlations between DAS-1 scores and criterion variables were MST (*r* = 0.65), TCD (*r* = 0.52), SKD (*r* = 0.43), and SCD (*r* = 0.60). According to the criteria of Evers et al. (2013), these are all excellent values except for SKD, which has a good criterion validity value.

**TABLE 4:** Distributional characteristics of the criterion variables.

				Number of
Variable	Range	Skewness	Kurtosis	Outliers
MST	1–5	0.49	–0.07	1
TCD	1–5	0.59	–0.23	8
SKD	1–4	0.55	0.07	0
SCD	1–5	-0.40	–0.39	10
Dissatisfaction	15–70	0.48	0.68	3
Impact	17–67	0.57	0.17	1
Social concern	7–34	0.94	0.87	4
Concern for the future	4–20	1.03	1.19	6
DQOL	45–186	0.74	1.01	2
BDI-II	0–49	1.44	1.92	5
SDS	23–70	0.69	–0.27	1
STAI-T	0–53	0.62	–0.46	0
STAI-S	1–57	1.72	7.07	1

The variable MST was dichotomized by the mean. The PMS (*N* = 143) and NMS (*N* = 61) categories were considered. ROC analysis was conducted taking into account the proportion (0.70) of patients in a PMS in this sample. The optimal cut-off score (50) for the DAS-1 was determined. Of our total sample, 150 subjects (73.5%) scored lower than 50 (better adjustment), and 54 subjects (26.5%) scored equal to or greater than 50 (poorer adjustment) according to the DAS-1. The AUC was 0.85. Efficacy indices for this cut-off were: sensitivity = 90.21% (95% CI: 85.28–93.92) specificity = 65.57% (95% CI: 89.35–99.95), overall efficiency = 82.84%. Correlation between categories (PMS and NMS) and classification on the DAS-1 (poorer/better adjustment; cut-off = 50) was φ = 0.58. Thus, 34% of the variability between PMS and NMS could be explained by the variability in the adjustment established by this cut-off.

Range, skewness, kurtosis, and number of outliers for the subscales (Dissatisfaction, Impact, Social concern, Concern for the future) and total DQOL are shown in Table 4. Among these, the following should be noted: the subscale Concern for the future has high skewness, high kurtosis and a high number of outliers. The Kolmogorov–Smirnov test was statistically significant for all the subscales, except for Dissatisfaction and DQOL. According to the criteria of Evers et al. (2013), the results showed excellent associations between the DAS-1 and the DQOL subscales Dissatisfaction (*r* = 0.77), Impact (*r* = 0.81), Social concern (*r* = 0.56), Concern for the future (*r* = 0.67), and between the DAS-1 and DQOL (*r* = 0.86). Better adjustment to T1D indicates better quality of life.

Properties of the score distributions for the instruments (BDI-II, SDS, STAI-T and STAI-S) are provided in Table 4. BDI-II and STAI-S distributions have skew >1, and they are more leptokurtic than the rest of the variables, highlighting the kurtosis (7.07) of STAI-S. The Kolmogorov–Smirnov test was statistically significant for all the scales. Excellent associations were found between the DAS-1 and BDI-II (*r* = 0.75), SDS (*r* = 0.70) STAI-T (*r* = 0.74), and STAI-S (*r* = 0.73). Better adjustment to T1D (lower DAS-1 scores) indicates a better emotional state (lower BDI, SDS, and STAI scores) and vice versa.

### Additional Construct Validity Evidence

Inadequate associations were found between the DAS-1 and the following clinical variables: Number of episodes per week of hypoglycemia (*r* = −0.08, *p* = 0.25) or hyperglycemia (*r* = 0.15, *p* < 0.05), Self-monitoring frequency (*r* = −0.12, *p* = 0.10), Age (*r* = 0.08, *p* = 0.27), and Number of years with diabetes (*r* = −0.03, *p* = 0.71). A statistically significant relationship was found between the degree of adjustment to T1D measured with the DAS-1 and the number of hyperglycemic episodes per week given the poor metabolic control of a large number of participants (60.4%). The remaining clinical variables did not reach statistical significance.

Differences in the DAS-1 score means in the two groups (Yes/No) established by the variables Other chronic diseases, Family, Partner, Work and Personal health support, and Diabetes complications were analyzed to obtain further evidence of construct validity. Means and Standard deviations of the DAS-1 scores for these variables in each group are shown in Table 1. This Table also shows the number and percentage of sample subjects in each group (Yes/No). Student’s *t*-test results comparing the DAS-1 score means in both groups for each variable are provided in Table 5. Results showed that the DAS-1 score means differed significantly (*p* < 0.05) in both groups (Yes/No) established by the variables Other chronic diseases and Family, Partner and Work support. No statistically significant differences were found in Diabetes complications and Personal health support. Hypothesis testing showed high power in all variables except for Diabetes complications and Partner support (<0.80). The size of the mean differences (*d*) on the DAS-1 was large for Family (1.17) and Personal health support (1.00), medium for Other chronic diseases (0.50) and Work support (0.50) and small for Partner support (0.33) and Diabetes complications (0.49).

**TABLE 5:** Results of the *t*-test on the DAS-1 scores in the criterion variables.

Variables	*t*	*df*	*p*	*d*	1-β	*95% CI*	
Other chronic diseases	2.80	201	<0.05	0.50	0.90	–9.07	–1.58
Family support	3.41	201	<0.05	1.17	0.95	–21.34	–5.70
Partner support	2.02	201	<0.05	0.33	0.51	–8.26	–0.09
Work support	2.99	92.26	<0.05	0.50	0.90	–10.37	–2.09
Personal health support	1.87	10.42	0.09	1.00	0.89	–24.48	2.06
Diabetes complications	1.98	44.74	0.05	0.49	0.76	–0.09	11.01

**d*: effect size; test power: *1−*β**; *CI*: confidence interval.*

Preliminary analysis of HbA_1c_% (*N* = 202) showed skew = 2.10, kurtosis = 6.46 and 9 outliers [Q3+1.5(IQR) = 10.45] in this sample. The HbA_1c_% groups are shown in Table 1. The Kruskal–Wallis test ($\chi_{2}^{2}=9.33$, *p* < 0.001) displayed significant differences between groups in the DAS-1 medians (38, 38, 55 in the sample, respectively). Multiple comparisons testing showed significant differences (*p* < 0.05) between groups (HbA_1c_% ≤ 7) and (7 < HbA_1c_% < 10) regarding extremely poor metabolic control (HbA_1c_% = 10). No significant differences (*p* = 0.97) were seen between groups (HbA_1c_% ≤ 7) and (7 < HbA_1c_% < 10). Subsequently, the DAS-1 detects differences in HbA_1c_, differentiating between those who had good, poor or extremely poor metabolic control.

Preliminary analysis of BMI (*N* = 199) showed skew = 0.23, kurtosis = 4.46 and 5 outliers [Q3+1.5(IQR) = 34.16] in this sample. The BMI scores had outliers, showing a highly leptokurtic distribution. The minimum outlier value for BMI was 34.16. After eliminating the two outliers in the DAS-1 scores (*N* = 197), the BMI groups are presented in Table 1. Kruskal–Wallis testing ($\chi_{2}^{2}=0.09$, *p* = 0.95) showed no statistically significant differences between these groups in the DAS-1 medians (38.5, 39, 42.5 in the sample, respectively).

## Discussion

The purpose of this study was to develop a scale to quantify adjustment to diabetes specifically in patients with T1D. This new instrument has shown adequate psychometric properties in this sample of T1D patients. The DAS-1 can be used to differentiate between good or poor adjustment to T1D using the optimal cut-off obtained (50), demonstrating its clinical utility. Using this new instrument, 26.5% of the subjects in this study were not well adjusted to diabetes. This figure is in line with a previous publication that situates the number of subjects in whom this phase is prolonged or who do not successfully adjust at around 30% (de Ridder et al., 2008).

The DAS-1 scores have also shown an adequate degree of validity. Factorial validity is supported by our findings affirming the acceptable fit of a one-factor structure adjustment to T1D. Scores also have excellent internal consistency and good test–retest reliability.

In addition to the results concerning the psychometric properties of the scale, the analysis of the relationship between the DAS-1 and other variables provides a series of data that should be mentioned. The association between the DAS-1 scale and the question “In general, what is your mood state?” (MST) is of note. One of the aspects that indicate the degree of the patient’s adjustment to the disease is mood (de Ridder et al., 2008). A negative perception indicates poor adjustment to diabetes. Accordingly, it is understood that the variability in the total DAS-1 scores explains much of the variability in the patient’s general mood perception (MST).

Criterion validity is also supported by an excellent/good association between the DAS-1 and the measure of negative emotions and quality of life. Thus, better adjustment to T1D (low DAS-1 scores), indicates a better emotional state (lower BDI, SDS and STAI scores), which is consistent with previous literature (Peyrot et al., 2005; Lloyd et al., 2010). High correlations with DQOL (total scores and Impact) were found. Higher scores on the DAS-1 (poorer adjustment to diabetes) are associated with higher scores on the DQOL (total scores and Impact), indicating poorer quality of life. This shows that high adjustment to T1D predicts high perceived quality of life (Malik and Koot, 2009).

The observed associations between the DAS-1 and several clinical variables display additional evidence of DAS-1 construct validity as a statistically significant relationship was found between DAS-1 scores and the number of hyperglycemic episodes per week. Poor metabolic control (60.4% of the participants in our study) indicates poor adjustment to T1D as has been noted in the scientific literature (Di Matteo, 2004; Ortiz, 2006; Sacco and Yanover, 2006; Fisher et al., 2014), and it has been suggested that it is difficult to achieve glycemic control without considering the factors that influence adjustment to diabetes.

Further evidence of the construct validity of the DAS-1 was analyzed by comparing the mean scores of the DAS-1 in groups defined by other clinical variables. It was found that people with family support, partner support or work support have better adjustment to T1D. This is also an important finding of this study, suggesting that these variables should be considered as possible protective factors that facilitate adjustment, contributing to reduce the impact of diabetes (Kovacs et al., 2013; Young-Hyman et al., 2016). Indeed, the DAS-1 total score showed a high association with the DQOL Impact subscale and total scores. Thus, as the literature demonstrates, the better the patient’s adjustment to the disease, the better the patient’s perceived well-being. Conversely, having additional chronic diseases increases DAS-1 scores, showing poorer adjustment (Malik and Koot, 2009).

Differences were found between the DAS-1 and HbA_1c_ levels. Higher DAS-1 scores predict higher HbA1c, differentiating between those with good, poor or extremely poor glycemic control, which represents further evidence of construct validity. This finding is consistent with previous literature (Trief et al., 1998), and provides new evidence differentiating not only among those who have good or poor glycemic control but also extremely poor glycemic control.

The results of this study support the conclusion that the DAS-1 is a good instrument to evaluate the adjustment to diabetes in persons with T1D. Although several instruments have been developed to assess this adjustment, no specific instruments are available to evaluate the adjustment to diabetes in the clinical population of adult T1D patients. The DAS-1 scale offers the following advantages: (a) it is a specific assessment instrument for adults with T1D; (b) it is a short instrument that is quick and easy to administer; and (c) it is based on the optimal cut-off.

The DAS-1 offers professionals who provide care to individuals with T1D a useful tool to quickly and reliably identify patients who adjust poorly to diabetes to prevent adverse consequences such as poor quality of life (Malik and Koot, 2009) or negative emotions that can lead to elevated depressive symptoms (Robertson et al., 2012). This is especially important as increased depressive symptoms are associated with an increase in the severity and number of diabetes complications (Egede, 2005). The relative risk of macrovascular complications among people with diabetes and depression is 2.5 times higher than those without this condition; the risk of microvascular complications is more than 11 times higher; almost 7 times higher for disability and almost 5 times higher for mortality (Von Korff et al., 2005). In addition, a high rate of depression is associated with low adherence to medical treatment of diabetes, resulting in a total health care cost that is 4.5 times higher than for those with diabetes without depression (Egede et al., 2002).

The need for psychosocial support in the care of patients with diabetes to optimize health outcomes and health-related quality of life is recognized by the American Diabetes Association [ADA] (2020) and recommended with the highest criteria (A). Use of the DAS-1 would allow patients to receive early intervention and suitable treatment. Specific intervention programs could be designed for these patients, and adequate psychosocial care could be provided by establishing protocols for adjustment to T1D to prevent the adverse consequences mentioned and to minimize the associated costs. These protocols should consider: the barriers encountered by patients in diabetes self-management or in their lifestyle (Rubin, 2005), negative emotions such as distress or depression (Anarte, 2004; Peyrot et al., 2005; Lloyd et al., 2010), the impact of diabetes on patients and their relatives (Young-Hyman et al., 2016), the use of appropriate diabetes coping strategies (Duangdao and Roesch, 2008; Jaser et al., 2012), and improving treatment adherence (Jaser et al., 2012).

Although the preliminary results obtained with the DAS-1 provide evidence of its reliability, validity and usefulness, further studies are needed and many issues remain to be addressed to improve our understanding, with particular regard to psychological factors as determinants in the adjustment to such diseases as diabetes (Martino et al., 2019). Use of the DAS-1 to assess psychological adjustment to diabetes may help predict risk in people with T1D by identifying individuals with low levels of adjustment to the disease.

## Data Availability Statement

The datasets generated for this study are not made publicly available as the patients have provided their informed consent for the collection of data for the research but not for its publication. Requests should be directed to anarte@uma.es.

## Ethics Statement

The studies involving human participants were reviewed and approved by the University of Málaga. The patients/participants provided their written informed consent to participate in this study.

## Author Contributions

MA, MC, MD-L, and MR contributed to conception and design of the study. MC organized the database. TR performed the statistical analysis. MA and TR wrote the first draft of the manuscript. All authors contributed to manuscript revision, read, and approved the submitted version.

## Conflict of Interest

The authors declare that the research was conducted in the absence of any commercial or financial relationships that could be construed as a potential conflict of interest.
